# Linking Plant Specialization to Dependence in Interactions for Seed Set in Pollination Networks

**DOI:** 10.1371/journal.pone.0078294

**Published:** 2013-10-30

**Authors:** Cristina Tur, Rocío Castro-Urgal, Anna Traveset

**Affiliations:** Department of Biodiversity and Conservation, Institut Mediterrani d'Estudis Avançats (CSIC-UIB). Esporles, Illes Balears, Spain; Lakehead University, Canada

## Abstract

Studies on pollination networks have provided valuable information on the number, frequency, distribution and identity of interactions between plants and pollinators. However, little is still known on the functional effect of these interactions on plant reproductive success. Information on the extent to which plants depend on such interactions will help to make more realistic predictions of the potential impacts of disturbances on plant-pollinator networks. Plant functional dependence on pollinators (all interactions pooled) can be estimated by comparing seed set with and without pollinators (i.e. bagging flowers to exclude them). Our main goal in this study was thus to determine whether plant dependence on current insect interactions is related to plant specialization in a pollination network. We studied two networks from different communities, one in a coastal dune and one in a mountain. For ca. 30% of plant species in each community, we obtained the following specialization measures: (i) linkage level (number of interactions), (ii) diversity of interactions, and (iii) closeness centrality (a measure of how much a species is connected to other plants via shared pollinators). Phylogenetically controlled regression analyses revealed that, for the largest and most diverse coastal community, plants highly dependent on pollinators were the most generalists showing the highest number and diversity of interactions as well as occupying central positions in the network. The mountain community, by contrast, did not show such functional relationship, what might be attributable to their lower flower-resource heterogeneity and diversity of interactions. We conclude that plants with a wide array of pollinator interactions tend to be those that are more strongly dependent upon them for seed production and thus might be those more functionally vulnerable to the loss of network interaction, although these outcomes might be context-dependent.

## Introduction

Pollination is a very important ecosystem service [1] because plants benefit from animal pollination for seed production. Nearly 85% of all flowering plants are pollinated by animals [2] and 35% of global crop production depends on pollinators [3]. Thus, the study of plant-pollinator interactions and its functional consequences for plant reproduction have long interested ecologists. In the last decades, pollination ecology has expanded from studies focused in single species and involving pairs of interactions to wide community studies involving entire networks of interactions [4]–7. Tools from network theory help to disentangle the structure and properties of these complex webs of interactions [8], [9]. This network approach revealed several interesting findings regarding pollination specialization/generalization patterns at community level. The frequency distribution of species generalization (i.e. number of links per species or linkage level) follows a power-law distribution or truncated power-law [10], i.e. there are many species with few interactions (specialists) and a few with many interactions (generalists or hubs). Specialist species tend to interact with proper subsets of the species that generalists interact with, thus leading to the broadly observed topological pattern of nestedness [11]. Interestingly, interactions are asymmetric: (i) specialized plants tend to have generalized pollinators and vice versa [12] and (ii) the strength of each interaction is not reciprocal, so that if one plant is much dependent on a pollinator, that pollinator is not dependent upon that plant [13], [14].

However, despite much information has been accumulated on the topology of pollination networks, studies linking network structure and functionality are still scarce [15]. The first studies have shown that network position of individual plants influences their fitness, individuals in central positions showing higher fitness than those in peripheral positions [16]. Moreover, recent studies have made important advances providing field estimates of the magnitude of species impacts and interaction strengths [17]. However, further research is needed to fill the existent gap of knowledge on the consequences of network links for plant reproductive success. This knowledge will help to determine the real plant functional dependence on such interactions and to make better predictions on how can they be affected by the loss of interactions.

Obviously, measuring the plant functional dependence in a per-interaction basis for all network links would require an enormous amount of fieldwork. Therefore, we propose a simplified approach consisting in measuring plant reproductive dependence on all pollinator interactions (i.e. pooling the effect of all pollinators). Dependence can be defined as the magnitude of seed set reduction when plant species are not pollinated by animals [3]. Highly dependent plants are those for which a high reduction in seed set occurs when pollinators are excluded, i.e. plants for which animal pollination is essential. In the present study, we ask: are the plants with more links in the networks those that in turn are more dependent upon pollinators for seed production? Specifically, we want to assess whether the degree of plant dependence on pollinators to set seeds is associated with: (i) total number of interactions (i.e. linkage level), (ii) diversity of interactions or (iii) topological position of each plant species within the plant-pollinator network (closeness centrality). These indices have been proposed as measures of specialization in pollination networks [18]. If the observed network links are contributing effectively to plant reproduction, we would expect number and diversity of interactions to positively influence seed set. Previous empirical studies have found a positive relationship between pollinator diversity and plant reproductive success [19]–[24]. Therefore, plants with high diversity and number of links in the networks may be those depending more strongly on pollinators. However, some specific studies [25] have found maximum reproductive success at intermediate levels of pollinator diversity, which suggests the existence of an optimal level of generalization. Moreover, plants in central positions in the network, i.e. highly connected to other plant species through shared pollinators, may experience a reduction in the amount of pollination received because of potential heterospecific deposition of pollen on stigmas by generalist pollinators [26]. Alternatively, thus, plants with a high dependence on animal pollinators to produce seeds might rely just on a few but effective interactions.

## Materials and Methods

### Ethics Statement

Servei de Protecció d’Espècies, Espais de Natura Balear (Conselleria d’Agricultura, Medi Ambient i Territori) and the military from Acar Puig Major/EVA n°7 (Ministry of Defence) provided permission to work at the study sites.

### Sampling Plant-pollinator Networks

The study was conducted in two different communities from Mallorca (Balearic Islands, Spain): (i) a dune marshland community at sea level located in the northeast of the island (Son Bosc, 39°46′28.11″N; 3°07′45.34″E; SB hereafter) and (ii) a high mountain shrub community at ca. 1100 m above sea level (Sa Coma de n’Arbona in Puig Major, 39°47′59.51″N; 2°47′07.81″E; PM hereafter). Both communities differ in plant species composition (Bray-Curtis binary dissimilarity among sites is 0.9) and flower abundances, being much higher in the coastal (mean ± SD: 31.51±145.58 flowers/m^2^ per species) than in the mountain community (2.30±5.893 flowers/m^2^ per species). We sampled plant-pollinator interactions in both communities during two consecutive flowering seasons (years 2009 and 2010), from April to July at SB and from May to August at PM. Sampling method consisted of time- fixed (3 min in SB and 5 min in PM) pollinator censuses on randomly-selected plant individuals of every species in bloom. During each census, we recorded: (i) taxonomic identity of plant species observed, (ii) taxonomic identity of insect flower-visitors observed (pollinators, hereafter) and (iii) number of flower visits made by each pollinator species, i.e. number of pollinator contacts with flower reproductive parts. When pollinators were not identified in the field they were captured for further identification by taxonomist experts. All plant species in bloom in the communities were sampled weekly at each site, between 10:00 am–5:00 pm on sunny and non-windy days. Weekly sampling effort was the same for all plant species in bloom regardless their abundance, although total census time accumulated throughout the sampling season differed across species, sites and years due to differences in plant species richness and flowering phenologies. In 2009, total census time was 42 h 18 min (SB) and 13 h 20 min (PM), while in 2010 it was 49 h 39 min (SB) and 38 h 15 min (PM).

For each study site, pollinator census data from the two years were pooled to construct a plant-pollinator weighted bipartite network. Plants and pollinators are nodes linked when an interaction between them was observed and each link has a specific weight depending on interaction frequency. These networks were represented by a quantitative interaction matrix *p x a*, where *p* is the number of plant species in the community, *a* is the number of pollinator species and the value in each matrix cell *n_ij_* is the interaction frequency measured as visits per flower per unit time made by pollinator *j* to plant species *i*. Interaction frequency is considered to be a good surrogate of total interaction effect of mutualist animals on plant reproduction [27], [28], [17]. As simple descriptors of these networks we calculated: (i) network size (S), i.e. number of plant nodes *x* number of pollinator nodes; (ii) total number of interactions; (iii) average number of interactions per species (I); (iv) interaction diversity (H_2_), i.e. Shannon’s diversity of interactions for the whole network; and (v) interaction evenness (E_2_), i.e. Shannon’s evenness measuring the heterogeneity in the frequency of interactions across the network (0 = uneven, 1 = uniform).

### Plant Specialization Level in Networks

For a subset of selected plant species from our networks (27 species in SB and 11 species in PM, see next section for details), we calculated linkage level (L), diversity of interactions (H) and closeness centrality (CC). These indices result from different ways of measuring species specialization level in networks, matching different concepts and aspects of specialization [18]. Linkage level (L) is the total number of interactions for each plant species. A complete list of the observed insect pollinators and their interaction frequencies can be found in Tables S2 and S3. Diversity of interactions (H) is the Shannon-Wiener diversity calculated as 
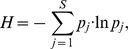
 where *p_j_* is the interaction frequency among pollinator *j* and plant species *i* relative to total interaction frequency of *i* (row sum) and *S* is the total number of plant *i*’s pollinators. Because diversity incorporates richness and evenness it can provide a much more accurate understanding of specialization, particularly when the number of flower visits is unevenly distributed across different pollinators. Closeness centrality (CC) was proposed as a measure of specialization in pollination networks [29] based on node position in the network. CC is the inverse of the average shortest distance between a focal plant species node and every other plant species nodes in a unipartite plant-plant network derived from the bipartite plant-pollinator network. In the unipartite network, two plant species are linked directly if they share at least one pollinator species. Therefore, CC measures the proximity of a plant species to other plant species. A plant is central when it has a high CC value which means is close to other plants in the network via shared pollinators. All indices were calculated using bipartite [30], [31] and sna packages [32] in R statistical programme version 2.15 [33]. Software Gephi 0.8 [34] was used for network drawings.

### Degree of Plant Dependence on Insect Pollination

For each study community, we evaluated the seed set -with and without pollinators- of several abundant and representative plant species. A total of 27 plant species were studied in SB, during 2009 and 2010, whereas 11 species were studied in PM in 2010. In both sites, the number of species selected (Table 1) represented ca. 30% of the entire plant assemblage, including 42% of all plant families present in SB and 35% in PM. These selected plant species covered the full range of specialization level in our networks (i.e. from specialist plants with one or two pollinators to generalist plants with more than 20 pollinators) and all were sampled a minimum observation time of 30 min in pollinator census. Two different treatments for each plant species studied were conducted: (i) Open pollination (OP), naturally pollinated flowers without manipulation and (ii) Pollinator exclusion (PE), in which flowers were covered with fine mesh bags that prevented insects visiting them but allowed wind- and self-pollination. Treatments started when plants had flowers at bud stage. Flowering branches or flower pedicels of each plant were marked, and flower units (flowers or inflorescences in the case of Asteraceae) were counted for each treatment. The number of flower units examined varied among individual plants and treatments depending on individual plant floral display and type of inflorescence (Table S1). Plants were monitored until fruits were almost mature, moment when bags were removed and fruits were collected. In the laboratory, fruits were dissected and viable seeds counted under the stereomicroscope when necessary. Mean seed set for each treatment was calculated as the total number of seeds produced per marked flower unit.

**Table 1:** Specialization indices obtained for plant species studied in each site and degree of plant dependence on insect pollination (IPD).

Site	Plant family	Plant species	Obs. time (min)	L	H	CC	IPD(%)
SB	Liliaceae	*Allium roseum*	50	11	1.50	0.87	79.72
SB	Liliaceae	*Asphodelus fistulosus*	135	12	1.94	0.92	0
SB	Scrophulariaceae	*Bellardia trixago*	71	4	0.93	0.78	0
SB	Gentianaceae	*Blackstonia perfoliata*	107	3	0.59	0.66	0
SB	Asteraceae	*Centaurea aspera*	120	17	1.66	0.88	46.42
SB	Gentianaceae	*Centaurium erythraea*	77	3	0.48	0.67	42.64
SB	Cistaceae	*Cistus salviifolius*	53	23	2.27	0.92	100
SB	Convulvulaceae	*Convolvulus althaeoides*	103	16	1.37	0.91	87.50
SB	Convulvulaceae	*Convolvulus arvensis*	113	25	1.63	0.95	87.03
SB	Asteraceae	*Crepis vesicaria*	67	15	2.14	0.92	97.74
SB	Apiaceae	*Daucus carota*	119	41	3.04	0.87	82.82
SB	Boraginaceae	*Echium sabulicola*	151	20	2.02	0.91	41.70
SB	Apiaceae	*Foeniculum vulgare*	42	10	1.78	0.78	61.31
SB	Asteraceae	*Helichrysum stoechas*	80	27	2.55	0.92	61.64
SB	Clusiaceae	*Hypochoeris achyrophorus*	68	11	2.02	0.86	96.43
SB	Asteraceae	*Hypericum perforatum*	80	9	1.66	0.80	19.74
SB	Fabaceae	*Lotus corniculatus*	147	18	2.38	0.89	100
SB	Fabaceae	*Lotus cytisoides*	89	9	1.43	0.88	100
SB	Fabaceae	*Medicago litoralis*	132	5	0.88	0.76	0
SB	Fabaceae	*Melilotus indica*	33	6	1.52	0.74	0
SB	Fabaceae	*Melilotus segettalis*	64	3	0.64	0.73	0
SB	Scrophulariaceae	*Parentucellia viscosa*	64	2	0.67	0.57	52.54
SB	Rosaceae	*Potentilla reptans*	86	28	2.62	0.96	98.96
SB	Asteraceae	*Scabiosa maritima*	120	24	1.87	0.95	78.45
SB	Caryophyllaceae	*Silene vulgaris*	70	3	0.36	0.77	96.77
SB	Lamiaceae	*Teucrium dunense*	92	28	2.08	0.94	63.84
SB	Scrophulariaceae	*Verbascum sinuatum*	101	11	1.49	0.78	85.02
PM	Caryophyllaceae	*Arenaria grandiflora*	75	8	1.75	0.73	77.02
PM	Asteraceae	*Bellium bellidioides*	135	13	2.20	0.86	75.81
PM	Asteraceae	*Carlina corymbosa*	80	18	1.86	0.82	96.72
PM	Asteraceae	*Crepis triasii*	85	14	2.12	0.89	94.68
PM	Rubiaceae	*Galium balearicum*	80	1	0	0.53	76.36
PM	Rubiaceae	*Galium cinereum*	85	2	0.28	0.70	25.61
PM	Cistaceae	*Helianthemum apenninum*	100	4	0.70	0.68	93.11
PM	Lamiaceae	*Rosmarinus officinalis*	45	9	1.39	0.79	42.40
PM	Asteraceae	*Santolina chamaecyparissus*	85	15	1.96	0.85	42.78
PM	Crassulaceae	*Sedum dasyphyllum*	90	8	1.84	0.82	89.90
PM	Lamiaceae	*Teucrium asiaticum*	135	13	1.93	0.74	65.25

Obs. time: observation time accumulated in pollinator censuses (min), L: linkage level, H: diversity of interactions, CC: closeness centrality.

Previous studies with crops [3] defined several levels of dependence on animal-mediated pollination by estimating the magnitude of seed set reduction comparing experiments with and without animal pollinators. Following the same approach, we calculated dependence on insect pollination (IPD) for each plant species as the percentage of open pollination seed set (SS_OP_) attributable to insect pollinator interactions (i.e. open pollination seed set excluding self-pollination and wind-pollination seed set, SS_OP_ - SS_PE_). Therefore, IPD ranges from 100 for plants which totally relied on pollinators for seed production (i.e. all seed set was a consequence of insect interactions), regardless of whether they produced many or few seeds, to 0 for plants that either selfed or were pollinated by wind. It is a useful index as it can be compared across different plant species and calculated using other measures of reproductive success different from seed set without losing meaning and interpretation. However, IPD cannot be considered as a measure of absolute plant species dependence on pollinators, as it may be contingent upon the current abiotic conditions, including resource availability, and we also need to consider the fact that plants may be pollen limited for several reasons [35].

### Data Analysis

To test the relationship between IPD and plant specialization level we first performed simple linear and quadratic regressions. We retained the regressions providing the best fit (R^2^) and lowest significance p-values (P). Variables were log-transformed when necessary to meet residuals’ normality assumption. To ensure that results in the mountain community (PM) were not caused by a low statistical power due to the relative small number of species, we bootstrapped the data (1000 times resampling with replacement) to increase sample size from 11 to 27 species (same number of species as in the larger community, SB). Regressions were repeated with each bootstrap and the number of significant regressions was calculated.

The presence of phylogenetic related plant species in the community can produce biases in regression analyses, thus we performed the same regressions with Generalized Estimating Equations (GEE) [36]. This method incorporates a correlation matrix of dependencies among observations in the modelling process. The correlation matrix is obtained from the phylogenetic tree of species in the community previously constructed with the free available software Phylocom 4.2 [37]. All phylogenetic analyses were done with function *compar.gee* from the ape package version 3.0–3 [38] implemented in R. Tree polytomies were resolved randomly with function multi2di.

## Results

Plant-pollinator networks studied had very different sizes and number of interactions. A total of 696 interactions between 80 plants and 162 insect species were recorded in SB and a total of 250 interactions between 34 plants and 92 insect in PM site (S_SB_ = 12960, S_PM_ = 8464). Both the average number (I_SB_ = 2.87, I_PM_ = 1.98) and the diversity of interactions per species (H_2 SB_ = 5.29, H_2 PM_ = 4.53) were higher in the coastal than in the mountain community, although the heterogeneity in interaction frequencies was similar in the two communities (E_2 SB_ = 0.80; E_2 PM_ = 0.82).

**Figure 1:**
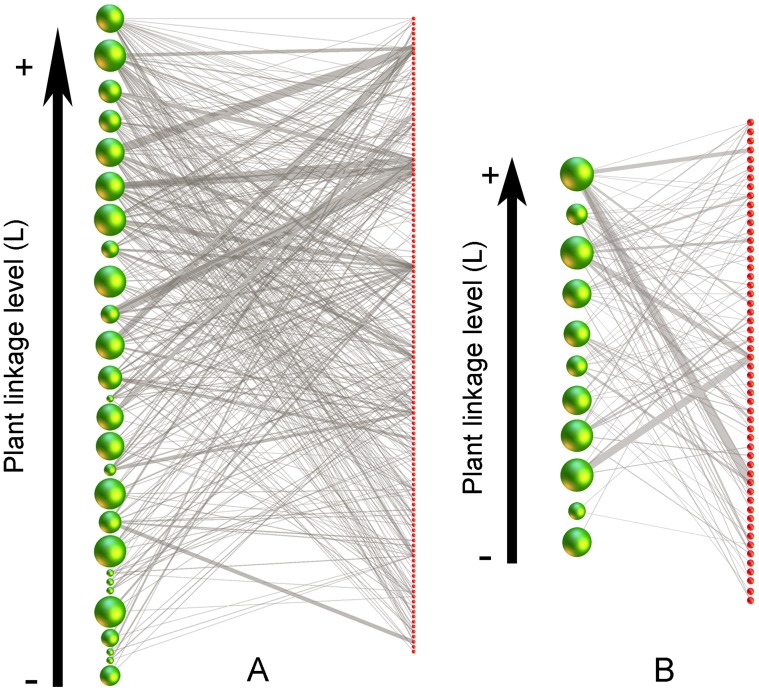
Plant dependence on interactions in pollination networks. Bipartite representation of networks only including plant species whose seed set was studied: (a) SB site (27 plants x 126 insects) and (b) PM site (11 plants x 54 insects). Green nodes represent plant species, red nodes represent pollinator species and links are weighted by interaction frequency (visits per flower/min). Plant nodes are ordered by linkage level (L) from the most specialist (bottom) to the most generalist (top). Within each network plant node size is proportional to the insect pollination dependence (IPD) (be aware size of nodes cannot be compared among subnetworks because they have been rescaled to fit in the figure). In SB network, the smallest green nodes are mainly concentrated in the bottom of the figure, indicating plants with a small linkage level were those with the lowest dependences on insect interactions. This trend is not observed in PM network where plants with just a few interactions (low L) were relatively highly dependent. Phylogenetic relationships between plants are not considered here.

**Figure 2:**
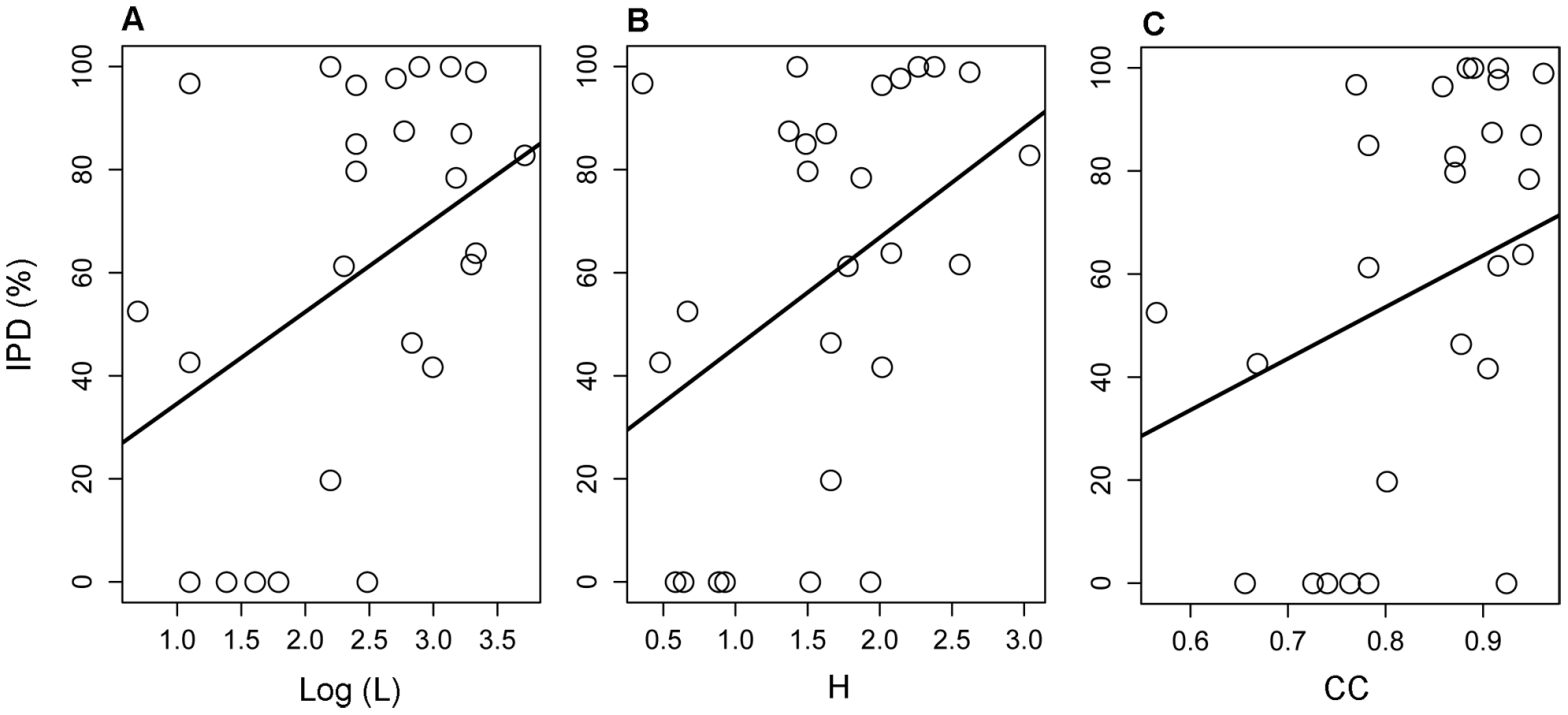
Relationships between plant dependence on insect pollination and plant specialization. Regressions obtained for the coastal community (SB). The degree of plant dependence on insect pollination (IPD) is the percentage of actual seed set attributed to pollinator interactions, i.e. excluding seed set caused by wind and self-pollination. Plant specialization is measured as: (a) linkage level (L), (b) diversity of interactions (H), and (c) closeness centrality (CC). Plotted lines are the fitted GEE models.

For the selected species (N_SB* = *_27, N_PM = _11), we report seed set obtained in each treatment in Table S1. Specialization indices and IPD are summarized in Table 1. Plants were less dependent on insects, on average, in SB (58.5±38.1%, mean ± sd) than in PM (70.9±24.2%). Results of the linear regressions between specialization indices and IPD are reported in Table 2. Results were consistent regardless phylogenetic relatedness among plants was controlled for or not. A significant relationship was found only in the larger and more heterogeneous coastal community (SB) (Fig. 1). In this community, highly dependent plants tended to have more links and a higher diversity of interactions in the network than plants little dependent on pollinators (Fig. 2a,b). Furthermore, plants in central positions within the network (high CC), because they were visited by generalist pollinators which in turn visited many other plant species, showed also higher dependencies than plants occupying peripheral network positions (Fig. 2c). In the smaller mountain community (PM), the relationships between IPD and all three measures of plant specialization were non-significant (Table 2). Increasing the sample size with bootstrapping methods did not produce different results in the simple linear regressions (Figure S1), thus reducing the probability of an effect of statistical power and suggesting that there might be an ecological cause behind the lack of a relationship in this community.

**Table 2:** Results for simple linear regression analyses (LM) and phylogenetic linear regression analysis using GEE in the coastal community (SB) (*dfP* = 11.33, phylogenetic degrees of freedom as defined in Paradis & Claude 2002 [36]) and in the mountain community (PM) (*dfP* = 5.7).

Response	Variable	Regression type	Site	Estimate	SE	t	*P*
IPD	log (L)	LM	SB	23.506	7.626	3.082	**0.005**
			PM	5.768	8.556	0.674	0.517
		GEE	SB	17.798	4.964	3.585	**0.005**
			PM	0.626	7.744	0.081	0.939
IPD	H	LM	SB	24.429	9.681	2.523	**0.018**
			PM	8.795	10.0570	0.875	0.404
		GEE	SB	21.332	6.602	3.231	**0.009**
			PM	3.962	8.987	0.440	0.683
IPD	CC	LM	SB	187.58	64.300	2.917	**0.007**
			PM	24.960	76.350	0.327	0.751
		GEE	SB	99.974	43.069	2.321	**0.044**
			PM	−103.92	68.468	−1.518	0.209

Significant relationships (*p-values* in bold numbers) between plant specialization and degree of plant dependence on insect pollination (IPD) were only found in one of the communities.

## Discussion

Our findings demonstrate that plants highly dependent on insects for pollination can be also those with high linkage levels, high diversity of interactions and occupying central positions in the network. We detected such relationship, however, only in one of the two communities studied (the largest, most diverse and most heterogeneous community), what suggests that the functional relationship is context-dependent and thus not consistent across all communities. If our results can be generalized to at least large pollination communities, it implies that plants dependent upon pollinators to seed set may ensure pollination by being generalists in the network, i.e. by attracting a wider array of pollinators. Generalization is considered to be a beneficial strategy, especially if pollinator abundances and interactions fluctuate across time, as found in most networks [39]–[42]. Moreover, there is evidence of positive effects of pollinator species richness and diversity on pollination services [19]–[21], [23], [24], indicating thus that a greater generalization tends to translate into greater reproductive success. Several possible mechanisms may explain the increase in seed production with increasing pollinator diversity [43]: (i) a sampling effect by which rich communities have more probabilities of including highly effective species or groups [44]; (ii) niche complementarity of pollinators, which occurs when species differ in their foraging patterns, for instance through space, time and/or environmental conditions [45], [22], [23], [24]; and (iii) functional facilitation, when the presence of a pollinator species enhances the performance of other species [46].

However, the functional relationship between plant dependence on insect pollination and generalization level might be weaker or simply absent in some contexts and communities. Previous studies have shown that, biodiversity has a higher impact on ecosystem functionality in naturally heterogeneous ecosystems - where niche complementarity can be most strongly expressed - and that resource heterogeneity may actually be required for a positive biodiversity-function relationship [47]. Our results are actually congruent with such findings, as the significant association between diversity of interactions and IPD was only found in the habitat with greater heterogeneity in flower-resource abundance and higher diversity of interactions. Interestingly, a theoretical approach [48] also suggested the diversity-function relationship can vary from negative to neutral to positive due to differences in effectiveness and abundance of pollinators. When the most abundant pollinators are also the most effective, it even may be beneficial for plants to be visited by a low diverse group of pollinators.

In addition, we found that topological position of a plant species within the network was also related to plant dependence on insect pollination. In individual-based one-mode networks, it has been recently found that plants occupying network central positions had higher fitness than those occupying peripheral positions, as chances of pollen outcrossing via shared pollinators with conspecific plants increase [16]. Following the same rationale, but turned into our species-based networks, we hypothesized that a high closeness centrality (CC) may imply negative effects for plant reproductive success because insects which are already visiting flowers of other plant species (i.e. generalist pollinators) may carry heterospecific pollen which could potentially interfere with conspecific pollen when deposited to stigmas [26], [49]. For this reason, dependent plants might benefit from not being central in networks. Interestingly, the opposite was found: highly dependent plants had a high connection to other plants through shared pollinators, suggesting that sharing pollinators with other plant species does not necessarily have negative competitive effects on reproductive success. However, this could be interpreted more as a result of generalist species occupying also central network positions [29] rather than an absence of negative interspecific pollen transfer effects. Quantitative information such as the frequency of interaction among each pollinator shared, the amount of interespecific pollen carried, or the frequency at which pollinators are shifting among plant species should be considered in order to adequately evaluate the potential competition for pollinators among plants [50].

Our study is only a first step in the understanding of the functional impact of network interactions on plant reproductive success. Most plant-pollinator network studies describe the pattern of interactions which take place in a community, but without measuring the real functional consequences of each of these interactions for plant reproduction. This occurs because quantifying the contribution of pollinator species to the reproductive output of plant species for each single network interaction would require a prohibitive amount of fieldwork. As far as we know, there is only one study conducted to date [17] which quantified the reciprocal impact of plants on pollinators and vice versa for five selected species of a network. Here, instead of measuring each per-interaction effect, we propose an alternative and simplifying approach based on measuring total-interactions effect on plant seed set, i.e. the percentage of actual seed set which depended on insect interactions. Obviously, using this approach precludes knowing to what extent each specific plant-pollinator link contributes to total plant seed set. High variability on the functional effect of each link should be expected, as flower-visitors vary in their pollination ability and effectiveness [51], [52]. Indeed, sometimes such network links may even not have a functional effect on plant reproduction because observed interactions do not translate always into true pollination events. For instance, our approach allowed us to detect some plants (n = 6) which had several interactions in the network (between two to 12) but with no real functional impact on seed set, because plants were self-pollinating. These observations highlight that inferring pollinator function directly from network data must be done with reserve. We further need to consider that the functional effect of such observed links for plant reproduction may change in time [53]–[55].

Linking network structure to community function is one of the forthcoming challenges in network ecology [56]. This kind of knowledge might be important in the future as it will permit, for instance, to make more realistic predictions of disturbance effects on plant-pollinator networks, to assess potential functional impacts of species loss or to help in species management decisions.

## Supporting Information

Figure S1 **Histograms showing the frequency of significance levels (P) obtained for the linear regressions performed using 1000 bootstraps of PM data with sample size n = 27.** Red dotted line indicates the boundary of P = 0.05. The percentage of cases resulting in a significant linear relationship among plant specialization indices (L: linkage level, H: diversity of interactions, C: closeness centrality) and degree of plant dependence on insect pollination (IPD) is very low in this community even when increasing sample size: 17.9%, 30% and 5.7% of significant regressions, respectively.
(TIF)

Table S1 **List of plant species selected for estimating seed production.** Here we indicate: study site, plant family, sample size as total number of plants and total number of flowers studied per treatment, mean seed set calculated as mean number of viable seeds per flower in each treatment.
(DOC)

Table S2 **List of plant-pollinator interactions observed in SB site for the 27 selected species.** Interaction frequency is the number of visits per flower per unit time made by each insect pollinator species.
(DOC)

Table S3 **List of plant-pollinator interactions observed in PM site for the 11 selected species.** Interaction frequency is the number of visits per flower per unit time made by each insect pollinator species.
(DOC)
