# Supplementary figures and images for: Linking Plant Specialization to Dependence in Interactions for Seed Set in Pollination Networks
Source: PLoS One. 2013 Oct 30;8(10):e78294. doi: 10.1371/journal.pone.0078294 (PMC3813576; doi:10.1371/journal.pone.0078294)

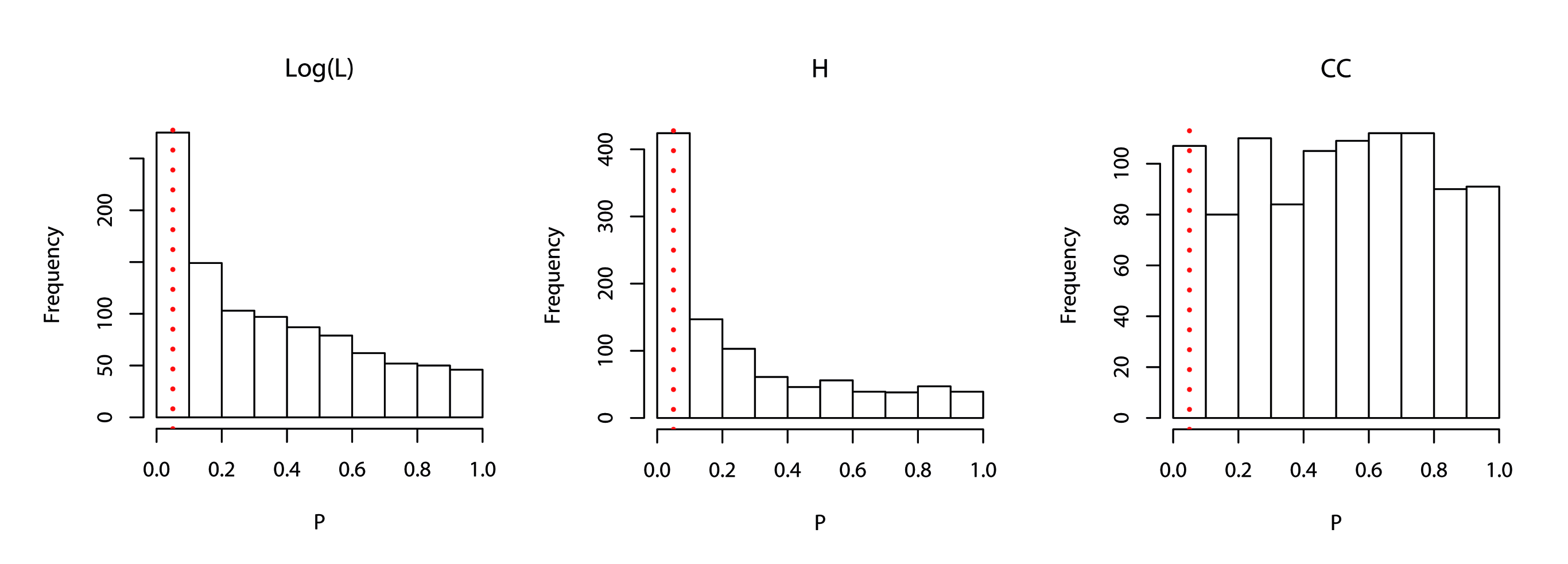

Supplement: Figure S1 — Histograms showing the frequency of significance levels (P) obtained for the linear regressions performed using 1000 bootstraps of PM data with sample size n = 27. Red dotted line indicates the boundary of P = 0.05. The percentage of cases resulting in a significant linear relationship among plant specialization indices (L: linkage level, H: diversity of interactions, C: closeness centrality) and degree of plant dependence on insect pollination (IPD) is very low in this community even when increasing sample size: 17.9%, 30% and 5.7% of significant regressions, respectively. (TIF) [file pone.0078294.s001.tif]
