# Supplementary material for: Linking Plant Specialization to Dependence in Interactions for Seed Set in Pollination Networks
Source: PLoS One. 2013 Oct 30;8(10):e78294. doi: 10.1371/journal.pone.0078294 (PMC3813576; doi:10.1371/journal.pone.0078294)
Supplement: Table S1 — List of plant species selected for estimating seed production. Here we indicate: study site, plant family, sample size as total number of plants and total number of flowers studied per treatment, mean seed set calculated as mean number of viable seeds per flower in each treatment. (DOC) [file pone.0078294.s002.doc]

**Table S1.**

|  |  |  | **OPEN POLLINATION** | | | | **POLLINATORS EXCLUSION** | | | |
| --- | --- | --- | --- | --- | --- | --- | --- | --- | --- | --- |
| **Site** | **Family** | **Species** | **Plants** | **Flowers** | **Seeds** | **Seed set** | **Plants** | **Flowers** | **Seeds** | **Seed set** |
| SB | Liliaceae | *Allium roseum* | 3 | 33 | 7 | 0.212 | 3 | 23 | 1 | 0.043 |
| SB | Liliaceae | *Asphodelus fistulosus* | 3 | 35 | 81 | 2.314 | 3 | 15 | 36 | 2.400 |
| SB | Scrophulariaceae | *Bellardia trixago* | 3 | 19 | 4786 | 251.895 | 3 | 16 | 4952 | 309.500 |
| SB | Gentianaceae | *Blackstonia perfoliata* | 3 | 101 | 12598 | 124.733 | 3 | 83 | 14028 | 169.012 |
| SB | Asteraceae | *Centaurea aspera ** | 3 | 3 | 7 | 2.333 | 3 | 4 | 5 | 1.250 |
| SB | Gentianaceae | *Centaurium erythraea* | 3 | 75 | 9623 | 128.307 | 3 | 78 | 5741 | 73.603 |
| SB | Cistaceae | *Cistus salviifolius* | 3 | 25 | 298 | 11.920 | 3 | 21 | 0 | 0.000 |
| SB | Convulvulaceae | *Convolvulus althaeoides* | 3 | 10 | 20 | 2.000 | 3 | 4 | 1 | 0.250 |
| SB | Convulvulaceae | *Convolvulus arvensis* | 3 | 24 | 20 | 0.833 | 3 | 37 | 4 | 0.108 |
| SB | Asteraceae | *Crepis vesicaria** | 3 | 38 | 761 | 20.026 | 3 | 31 | 14 | 0.452 |
| SB | Apiaceae | *Daucus carota* | 3 | 10845 | 7004 | 0.646 | 3 | 9400 | 1040 | 0.111 |
| SB | Boraginaceae | *Echium sabulicola* | 3 | 23 | 37 | 1.609 | 3 | 16 | 15 | 0.938 |
| SB | Apiaceae | *Foeniculum vulgare* | 4 | 700 | 718 | 1.026 | 3 | 917 | 364 | 0.397 |
| SB | Asteraceae | *Helichrysum stoechas ** | 3 | 153 | 866 | 5.660 | 3 | 175 | 380 | 2.171 |
| SB | Clusiaceae | *Hypericum perforatum* | 3 | 253 | 118 | 0.466 | 3 | 203 | 76 | 0.374 |
| SB | Asteraceae | *Hypochoeris achyrophorus ** | 3 | 7 | 56 | 8.000 | 3 | 7 | 2 | 0.286 |
| SB | Fabaceae | *Lotus corniculatus* | 3 | 18 | 214 | 11.889 | 3 | 24 | 0 | 0.000 |
| SB | Fabaceae | *Lotus cytisoides* | 3 | 15 | 20 | 1.333 | 3 | 15 | 0 | 0.000 |
| SB | Fabaceae | *Medicago litorales* | 3 | 28 | 37 | 1.321 | 3 | 49 | 200 | 4.082 |
| SB | Fabaceae | *Melilotus indica* | 3 | 127 | 43 | 0.339 | 3 | 154 | 119 | 0.773 |
| SB | Fabaceae | *Melilotus segettalis* | 3 | 97 | 97 | 1.000 | 3 | 112 | 152 | 1.357 |
| SB | Scrophulariaceae | *Parentucellia viscosa* | 3 | 55 | 4867 | 88.491 | 3 | 77 | 3234 | 42.000 |
| SB | Rosaceae | *Potentilla reptans* | 3 | 5 | 481 | 96.200 | 3 | 3 | 3 | 1.000 |
| SB | Asteraceae | *Scabiosa maritima ** | 3 | 3 | 181 | 60.333 | 3 | 3 | 39 | 13.000 |
| SB | Caryophyllaceae | *Silene vulgaris* | 3 | 46 | 374 | 8.130 | 3 | 38 | 10 | 0.263 |
| SB | Lamiaceae | *Teucrium dunense ** | 3 | 24 | 426 | 17.750 | 3 | 31 | 199 | 6.419 |
| SB | Scrophulariaceae | *Verbascum sinuatum* | 3 | 97 | 1160 | 11.959 | 3 | 77 | 138 | 1.792 |

*For these species number of flowers are inflorescences

**Table S1** *(cont.)*

|  |  |  | **OPEN POLLINATION** | | | | **POLLINATORS EXCLUSION** | | | |
| --- | --- | --- | --- | --- | --- | --- | --- | --- | --- | --- |
| **Site** | **Family** | **Species** | **Plants** | **Flowers** | **Seeds** | **Seed set** | **Plants** | **Flowers** | **Seeds** | **Seed set** |
| PM | Caryophyllaceae | *Arenaria grandiflora* | 2 | 8 | 22 | 2.750 | 3 | 19 | 12 | 0.632 |
| PM | Asteraceae | *Bellium bellidioides ** | 4 | 19 | 576 | 30.316 | 2 | 18 | 132 | 7.333 |
| PM | Asteraceae | *Carlina corymbosa ** | 3 | 15 | 687 | 45.800 | 3 | 14 | 21 | 1.500 |
| PM | Asteraceae | *Crepis triasii ** | 3 | 21 | 1397 | 66.524 | 2 | 13 | 46 | 3.538 |
| PM | Rubiaceae | *Galium balearicum* | 2 | 120 | 64 | 0.533 | 2 | 191 | 24 | 0.126 |
| PM | Rubiaceae | *Galium cinereum* | 2 | 381 | 110 | 0.289 | 4 | 390 | 84 | 0.215 |
| PM | Cistaceae | *Helianthemum apenninum* | 5 | 18 | 220 | 12.222 | 4 | 19 | 16 | 0.842 |
| PM | Lamiaceae | *Rosmarinus officinalis* | 3 | 55 | 76 | 1.382 | 3 | 54 | 43 | 0.796 |
| PM | Asteraceae | *Santolina chamaecyparissus ** | 4 | 40 | 329 | 8.225 | 4 | 34 | 160 | 4.706 |
| PM | Crassulaceae | *Sedum dasyphyllum* | 3 | 59 | 483 | 8.186 | 4 | 127 | 105 | 0.827 |
| PM | Lamiaceae | *Teucrium asiaticum* | 4 | 81 | 238 | 2.938 | 3 | 48 | 49 | 1.021 |

*For these species number of flowers are inflorescences.
