# Supplementary material for: Linking Plant Specialization to Dependence in Interactions for Seed Set in Pollination Networks
Source: PLoS One. 2013 Oct 30;8(10):e78294. doi: 10.1371/journal.pone.0078294 (PMC3813576; doi:10.1371/journal.pone.0078294)
Supplement: Table S2 — List of plant-pollinator interactions observed in SB site for the 27 selected species. Interaction frequency is the number of visits per flower per unit time made by each insect pollinator species. (DOC) [file pone.0078294.s003.doc]

**Table S2.**

| **PLANT** | | **INSECT POLLINATOR** | | **Interaction frequency** |
| --- | --- | --- | --- | --- |
| **Family** | **Species name** | **Family** | **Species or morphospecies name** |
| Liliaceae | *Allium roseum* | Apidae | *Apis mellifera* | 0.52 |
|  |  | Anthomyiidae | *Delia platura* | 0.04 |
|  |  | Bibionidae | *Dilophus antipedalis* | 2.22 |
|  |  | Formicidae | *Linepithema humile* | 0.42 |
|  |  | Nitidulidae | *Meligethes* sp. | 0.72 |
|  |  | Apidae | *Osmia caerulescens* | 0.06 |
|  |  | Apidae | *Osmia latreillei* | 0.12 |
|  |  | Chrysomelidae | *Spermophagus* sp. | 0.30 |
|  |  | Vespidae | *Stenodynerus f. fastidiosissimus* | 0.04 |
|  |  | Formicidae | *Tapinoma madeirense* | 0.04 |
|  |  | Curculionidae | *Tychius aureolus* | 0.04 |
| Liliaceae | *Asphodelus fistulosus* | Apidae | *Apis mellifera* | 0.36 |
|  |  | Apidae | *Ceratina* sp. *(cucurbitina + dellatorreana)* | 0.07 |
|  |  | Bibionidae | *Dilophus antipedalis* | 0.12 |
|  |  | Eurytomidae | Eurytomidae sp. | 0.05 |
|  |  | Apidae | *Lasioglossum griseolum* | 0.01 |
|  |  | Apidae | *Lasioglossum villosulum* | 0.01 |
|  |  | Formicidae | *Linepithema humile* | 0.03 |
|  |  | Nitidulidae | *Meligethes* sp. | 0.15 |
|  |  | Mordellidae | *Mordellistena* sp. | 0.01 |
|  |  | Lygaeidae | *Nysius cymoides* | 0.01 |
|  |  | Chrysomelidae | *Spermophagus* sp. | 0.01 |
|  |  | Rhinophoridae | *Stevenia deceptoria* | 0.01 |

**Table S2** (*cont*.)

| **PLANT** | | **INSECT POLLINATOR** | | **Interaction frequency** |
| --- | --- | --- | --- | --- |
| **Family** | **Species name** | **Family** | **Species or morphospecies name** |
| Scrophulariaceae | *Bellardia trixago* | Apidae | *Lasioglossum malachurum* | 0.01 |
|  |  | Nitidulidae | *Meligethes* sp. | 0.18 |
|  |  | Apidae | *Osmia aurulenta* | 0.08 |
|  |  | Dasytidae | *Psilotrix* sp. *(illustris + cyaneus + aureolus)* | 0.06 |
| Gentianaceae | *Blackstonia perfoliata* | Curculionidae | *Aulacobaris* sp. | 0.06 |
|  |  | Vespidae | *Eumenes c. coarctatus* | 0.02 |
|  |  | Syrphidae | *Sphaerophoria* sp. *(scripta + rueppellii)* | 0.03 |
| Asteraceae | *Centaurea aspera* | - | Acari sp. | 0.12 |
|  |  | Apidae | *Apis mellifera* | 0.98 |
|  |  | Braconidae | Braconidae sp2 | 0.01 |
|  |  | Apidae | *Halictus* sp. *(scabiosae + fulvipes)* | 2.81 |
|  |  | Apidae | *Lasioglossum albocinctum* | 0.01 |
|  |  | Formicidae | *Linepithema humile* | 0.19 |
|  |  | Apidae | *Megachile apicalis* | 0.08 |
|  |  | Apidae | *Megachile pilidens* | 0.03 |
|  |  | Nitidulidae | *Meligethes* sp. | 0.15 |
|  |  | Mordellidae | *Mordellistena* sp. | 0.02 |
|  |  | Oedemeridae | *Oedemera caudata* | 0.02 |
|  |  | Oedemeridae | *Oedemera flavipes* | 0.01 |
|  |  | Oedemeridae | *Oedemera simplex* | 0.03 |
|  |  | Apidae | *Osmia latreillei* | 0.01 |
|  |  | Lycenidae | *Polyommatus icarus* | 0.06 |
|  |  | Apidae | *Rhodanthidium septemdentatum* | 0.05 |
|  |  | Nymphalidae | *Vanessa carduii* | 0.01 |

**Table S2** (*cont*.)

| **PLANT** | | **INSECT POLLINATOR** | | **Interaction frequency** |
| --- | --- | --- | --- | --- |
| **Family** | **Species name** | **Family** | **Species or morphospecies name** |
| Gentianaceae | *Centaurium erythraea* | Apidae | *Ceylalictus variegatus* | 0.03 |
|  |  | Syrphidae | *Helophilus trivittatus* | 0.09 |
|  |  | Syrphidae | *Sphaerophoria* sp. *(scripta + rueppellii)* | 0.03 |
| Cistaceae | *Cistus salviifolius* | - | Acari sp. | 0.30 |
|  |  | Apidae | *Andrena* subgen. *Micrandrena* | 0.02 |
|  |  | Dermestidae | *Anthrenus miniopictus* | 0.02 |
|  |  | Dermestidae | *Anthrenus pimpinellae* | 0.02 |
|  |  | Apidae | *Apis mellifera* | 0.36 |
|  |  | Chrysomelidae | *Bruchidius* sp1 | 0.09 |
|  |  | Chrysomelidae | Bruchidae sp3 | 0.02 |
|  |  | Milichiidae | *Desmometopa m-nigrum* | 0.02 |
|  |  | Malachiidae | *Ebaeua apendiculatus* | 0.04 |
|  |  | Apidae | *Eucera oraniensis* | 0.28 |
|  |  | Sciomyzidae | *Euthycera alaris* | 0.02 |
|  |  | Tenebrionidae | *Isomira* sp. | 0.08 |
|  |  | Apidae | *Lasioglossum prasinum* | 0.04 |
|  |  | Formicidae | *Linepithema humile* | 0.17 |
|  |  | Nitidulidae | *Meligethes* sp. | 0.91 |
|  |  | Mordellidae | *Mordellistena* sp. | 0.02 |
|  |  | Stratiomyidae | *Nemotelus pantherinus* | 0.04 |
|  |  | Oedemeridae | *Oedemera flavipes* | 0.09 |
|  |  | Cetoniidae | *Oxythyrea funesta* | 0.08 |
|  |  | Dasytidae | *Psilotrix* sp. *(illustris + cyaneus + aureolus)* | 0.04 |
|  |  | Apidae | *Rhodanthidium septemdentatum* | 0.04 |
|  |  | Chrysomelidae | *Spermophagus* sp. | 0.26 |
|  |  | Rhinophoridae | *Stevenia deceptoria* | 0.02 |

**Table S2** (*cont*.)

| **PLANT** | | **INSECT POLLINATOR** | | **Interaction frequency** |
| --- | --- | --- | --- | --- |
| **Family** | **Species name** | **Family** | **Species or morphospecies name** |
| Convolvulaceae | *Convolvulus althaeoides* | Apidae | *Apis mellifera* | 0.06 |
|  |  | Apidae | *Bombus terrestris* | 0.23 |
|  |  | Pieridae | *Colias croceus* | 0.03 |
|  |  | Malachiidae | *Ebaeua apendiculatus* | 0.01 |
|  |  | Syrphidae | *Helophilus trivittatus* | 0.01 |
|  |  | Nitidulidae | *Meligethes* sp. | 0.13 |
|  |  | Stratiomyidae | *Nemotelus pantherinus* | 0.01 |
|  |  | Oedemeridae | *Oedemera caudata* | 0.01 |
|  |  | Oedemeridae | *Oedemera flavipes* | 0.01 |
|  |  | Oedemeridae | *Oedemera simplex* | 0.06 |
|  |  | Dasytidae | *Psilotrix* sp. *(illustris + cyaneus + aureolus)* | 0.07 |
|  |  | Apidae | *Rhodanthidium septemdentatum* | 0.05 |
|  |  | Chrysomelidae | *Spermophagus* sp. | 1.83 |
|  |  | Calliphoridae | *Stomorhina lunata* | 0.01 |
|  |  | Curculionidae | *Tychius aureolus* | 0.01 |
|  |  | Nymphalidae | *Vanessa carduii* | 0.01 |

**Table S2** (*cont*.)

| **PLANT** | | **INSECT POLLINATOR** | | **Interaction frequency** |
| --- | --- | --- | --- | --- |
| **Family** | **Species name** | **Family** | **Species or morphospecies name** |
| Convolvulaceae | *Convolvulus arvensis* | - | Acari sp. | 0.04 |
|  |  | Apidae | *Andrena* sp1 *(ovatula + fulvipes)* | 0.04 |
|  |  | Apidae | *Ceratina* sp. *(cucurbitina + dellatorreana)* | 0.22 |
|  |  | Apidae | *Ceylalictus variegatus* | 0.04 |
|  |  | Cerambicidae | *Chlorophorus trifasciatus* | 0.01 |
|  |  | Pieridae | *Colias croceus* | 0.04 |
|  |  | Malachiidae | *Colotes maculatus* | 0.01 |
|  |  | Syrphidae | *Eristalinus aeneus* | 0.03 |
|  |  | Apidae | *Lasioglossum gemmeus* | 0.02 |
|  |  | Apidae | *Lasioglossum griseolum* | 0.02 |
|  |  | Apidae | *Lasioglossum malachurum* | 0.06 |
|  |  | Apidae | *Lasioglossum minutissimum* | 0.01 |
|  |  | Apidae | *Lasioglossum villosulum* | 0.02 |
|  |  | Nitidulidae | *Meligethes* sp. | 0.16 |
|  |  | Mordellidae | *Mordellistena* sp. | 0.04 |
|  |  | Stratiomyidae | *Nemotelus pantherinus* | 0.09 |
|  |  | Oedemeridae | *Oedemera caudata* | 0.02 |
|  |  | Oedemeridae | *Oedemera flavipes* | 0.02 |
|  |  | Oedemeridae | *Oedemera simplex* | 0.01 |
|  |  | Dasytidae | *Psilotrix* sp. *(illustris + cyaneus + aureolus)* | 0.03 |
|  |  | Cantharidae | *Rhagonycha fulva* | 0.02 |
|  |  | Chrysomelidae | *Spermophagus* sp. | 1.66 |
|  |  | Syrphidae | *Sphaerophoria* sp. *(scripta + rueppellii)* | 0.20 |
|  |  | Calliphoridae | *Stomorhina lunata* | 0.01 |
|  |  | Formicidae | *Tapinoma madeirense* | 0.01 |

**Table S2** (*cont*.)

| **PLANT** | | **INSECT POLLINATOR** | | **Interaction frequency** |
| --- | --- | --- | --- | --- |
| **Family** | **Species name** | **Family** | **Species or morphospecies name** |
| Asteraceae | *Crepis vesicaria* | Apidae | *Andrena* subgen. *Micrandrena* | 0.03 |
|  |  | Byrrhidae | Byrrhidae sp. | 0.03 |
|  |  | Apidae | *Eucera oraniensis* | 0.75 |
|  |  | Syrphidae | *Helophilus trivittatus* | 0.09 |
|  |  | Nitidulidae | *Meligethes* sp. | 0.40 |
|  |  | Mordellidae | *Mordellistena* sp. | 0.01 |
|  |  | Stratiomyidae | *Nemotelus pantherinus* | 0.07 |
|  |  | Oedemeridae | *Oedemera caudata* | 0.07 |
|  |  | Oedemeridae | *Oedemera flavipes* | 0.06 |
|  |  | Apidae | *Osmia latreillei* | 0.12 |
|  |  | Dasytidae | *Psilotrix* sp. *(illustris + cyaneus + aureolus)* | 0.30 |
|  |  | Cantharidae | *Rhagonycha fulva* | 0.01 |
|  |  | Chrysomelidae | *Spermophagus* sp. | 0.18 |
|  |  | Syrphidae | *Sphaerophoria* sp. *(scripta + rueppellii)* | 0.03 |
|  |  | Nymphalidae | *Vanessa carduii* | 0.07 |

**Table S2** (*cont*.)

| **PLANT** | | **INSECT POLLINATOR** | | **Interaction frequency** |
| --- | --- | --- | --- | --- |
| **Family** | **Species name** | **Family** | **Species or morphospecies name** |
| Umbelliferae | *Daucus carota* | - | Acari sp. | 0.42 |
|  |  | Dermestidae | *Anthrenus* sp. | 0.13 |
|  |  | Braconidae | Braconidae sp1 | 0.03 |
|  |  | Ceratopogonidae | Ceratopogonidae sp. | 0.01 |
|  |  | Apidae | *Ceylalictus variegatus* | 0.63 |
|  |  | Chironomidae | Chironomidae sp. | 0.15 |
|  |  | Cerambicidae | *Chlorophorus trifasciatus* | 0.64 |
|  |  | Coccinelidae | *Coccinella septempunctata* | 0.45 |
|  |  | Coccinellidae | *Coccinella undecimpunctata* | 0.03 |
|  |  | Syrphidae | *Eristalinus aeneus* | 0.45 |
|  |  | Syrphidae | *Eristalis arbustorum* | 0.42 |
|  |  | Syrphidae | *Eristalinus sepulchralis* | 0.13 |
|  |  | Gasteruptiidae | *Gasteruption undulatum* | 0.26 |
|  |  | Apidae | *Halictus* sp. *(scabiosae + fulvipes)* | 0.42 |
|  |  | Chrysididae | *Holopyga fervida* | 1.16 |
|  |  | Apidae | *Hylaeus* sp. *(clypearis + trinotatus + signatus + variegatus)* | 1.09 |
|  |  | Apidae | *Lasioglossum gemmeus* | 0.13 |
|  |  | Miridae | *Lepydargyrus ancorifer* | 0.03 |
|  |  | Formicidae | *Linepithema humile* | 1.89 |
|  |  | Tiphiidae | *Meria tripunctata* | 1.54 |
|  |  | Mordellidae | *Mordellistena* sp. | 1.05 |
|  |  | Stratiomyidae | *Nemotelus pantherinus* | 0.29 |
|  |  | Muscidae | *Neomyia cornicina* | 0.49 |
|  |  | Anthicidae | *Notoxus monoceros* | 0.03 |
|  |  | Oedemeridae | *Oedemera caudata* | 0.47 |
|  |  | Oedemeridae | *Oedemera simplex* | 0.38 |
|  |  | Cetoniidae | *Oxythyrea funesta* | 0.03 |

**Table S2** (*cont*.)

| **PLANT** | | **INSECT POLLINATOR** | | **Interaction frequency** |
| --- | --- | --- | --- | --- |
| **Family** | **Species name** | **Family** | **Species or morphospecies name** |
| Umbelliferae | *Daucus carota* | Agromyzidae | *Phytomyza* sp. | 0.01 |
|  |  | Vespidae | *Polistes* sp. *(dominulus + gallicus)* | 1.46 |
|  |  | Sphecidae | *Prionyx kirbii* | 0.03 |
|  |  | Muscidae | *Pyrellia vivida* | 0.03 |
|  |  | Cantharidae | *Rhagonycha fulva* | 2.45 |
|  |  | Sarcophagidae | *Sarcophaga villeneuveana* | 0.25 |
|  |  | Sarcophagidae | *Senotainia tricuspis* | 0.04 |
|  |  | Chrysomelidae | *Spermophagus* sp. | 1.15 |
|  |  | Syrphidae | *Sphaerophoria* sp. *(scripta + rueppellii)* | 0.25 |
|  |  | Rhinophoridae | *Stevenia deceptoria* | 0.95 |
|  |  | Syrphidae | *Syritta pipiens* | 0.35 |
|  |  | Formicidae | *Tapinoma madeirense* | 0.55 |
|  |  | Therevidae | *Thereva spiloptera* | 0.13 |
|  |  | Curculionidae | *Tychius aureolus* | 0.43 |

**Table S2** (*cont*.)

| **PLANT** | | **INSECT POLLINATOR** | | **Interaction frequency** |
| --- | --- | --- | --- | --- |
| **Family** | **Species name** | **Family** | **Species or morphospecies name** |
| Boraginaceae | *Echium sabulicola* | Apidae | *Amegilla balearica* | 0.50 |
|  |  | Apidae | *Amegilla quadrifasciata* | 0.13 |
|  |  | Apidae | *Ceratina* sp. *(cucurbitina + dellatorreana)* | 1.14 |
|  |  | Apidae | *Ceylalictus variegatus* | 0.01 |
|  |  | Bibionidae | *Dilophus antipedalis* | 0.02 |
|  |  | Curculionidae | *Gymnetron* sp. | 0.01 |
|  |  | Apidae | *Hoplitis adunca* | 0.03 |
|  |  | Apidae | *Hoplitis benoisti* | 0.01 |
|  |  | Apidae | *Lasioglossum gemmeus* | 0.03 |
|  |  | Apidae | *Lasioglossum griseolum* | 0.02 |
|  |  | Miridae | *Lepydargyrus ancorifer* | 0.01 |
|  |  | Nitidulidae | *Meligethes* sp. | 0.48 |
|  |  | Anthocoridae | *Orius niger* | 0.10 |
|  |  | Apidae | *Osmia adunca* | 0.05 |
|  |  | Apidae | *Osmia aurulenta* | 0.17 |
|  |  | Apidae | *Osmia caerulescens* | 0.33 |
|  |  | Apidae | *Osmia versicolor* | 0.07 |
|  |  | Dasytidae | *Psilotrix* sp. *(illustris + cyaneus + aureolus)* | 0.01 |
|  |  | Apidae | *Rhodanthidium septemdentatum* | 0.04 |
|  |  | Nymphalidae | *Vanessa carduii* | 0.01 |

**Table S2** (*cont*.)

| **PLANT** | | **INSECT POLLINATOR** | | **Interaction frequency** |
| --- | --- | --- | --- | --- |
| **Family** | **Species name** | **Family** | **Species or morphospecies name** |
| Umbelliferae | *Foeniculum vulgare* | Crabronidae | *Bembix occulata* | 1.19 |
|  |  | Syrphidae | *Eristalinus sepulchralis* | 0.24 |
|  |  | Apidae | *Lasioglossum gemmeus* | 0.43 |
|  |  | Rhinophoridae | *Phyto melanocephala* | 1.19 |
|  |  | Vespidae | *Polistes* sp. *(dominulus + gallicus)* | 4.88 |
|  |  | Pteromalidae | Pteromalidae sp. | 0.02 |
|  |  | Sarcophagidae | *Sarcophaga* sp. | 1.19 |
|  |  | Sarcophagidae | *Sarcophaga unicurva* | 0.19 |
|  |  | Sarcophagidae | *Sarcophaga villeneuveana* | 0.36 |
|  |  | Chrysomelidae | *Spermophagus* sp. | 2.24 |

**Table S2** (*cont*.)

| **PLANT** | | **INSECT POLLINATOR** | | **Interaction frequency** |
| --- | --- | --- | --- | --- |
| **Family** | **Species name** | **Family** | **Species or morphospecies name** |
| Asteraceae | *Helichrysum stoechas* | - | Acari sp. | 0.88 |
|  |  | Apidae | *Andrena* subgen. *Micrandrena* | 0.25 |
|  |  | Apidae | *Andrena* sp1 *(ovatula + fulvipes)* | 0.19 |
|  |  | Apidae | *Andrena* sp3 | 0.31 |
|  |  | Apidae | *Apis mellifera* | 0.19 |
|  |  | Blattodea | Blattodea sp. | 0.04 |
|  |  | Byrrhidae | Byrrhidae sp. | 0.01 |
|  |  | Cerambicidae | *Chlorophorus trifasciatus* | 0.85 |
|  |  | Muscidae | *Coenosia tigrina* | 0.04 |
|  |  | Apidae | *Colletes abeillei* | 1.54 |
|  |  | Apidae | *Halictus* sp. *(scabiosae + fulvipes)* | 0.19 |
|  |  | Apidae | *Heriades rubicolus* | 0.19 |
|  |  | Apidae | *Lasioglossum gemmeus* | 0.06 |
|  |  | Apidae | *Lasioglossum minutissimum* | 0.19 |
|  |  | Miridae | *Lepydargyrus ancorifer* | 0.04 |
|  |  | Gryllidae | *Melanogrillus desertus* | 0.01 |
|  |  | Nitidulidae | *Meligethes* sp. | 0.04 |
|  |  | Stratiomyidae | *Nemotelus pantherinus* | 2.63 |
|  |  | Muscidae | *Neomyia cornicina* | 0.19 |
|  |  | Oedemeridae | *Oedemera caudata* | 0.19 |
|  |  | Oedemeridae | *Oedemera flavipes* | 0.05 |
|  |  | Dasytidae | *Psilotrix* sp. *(illustris + cyaneus + aureolus)* | 0.04 |
|  |  | Cantharidae | *Rhagonycha fulva* | 0.83 |
|  |  | Sarcophagidae | *Sarcophaga villeneuveana* | 0.24 |
|  |  | Chrysomelidae | *Spermophagus* sp. | 0.09 |
|  |  | Rhinophoridae | *Stevenia deceptoria* | 0.28 |
|  |  | Syrphidae | *Syritta pipiens* | 0.06 |

**Table S2** (*cont*.)

| **PLANT** | | **INSECT POLLINATOR** | | **Interaction frequency** |
| --- | --- | --- | --- | --- |
| **Family** | **Species name** | **Family** | **Species or morphospecies name** |
| Guttiferae | *Hypericum perforatum* | Apidae | *Andrena* sp1 *(ovatula + fulvipes)* | 0.06 |
|  |  | Apidae | *Apis mellifera* | 0.01 |
|  |  | Apidae | *Ceratina* sp. *(cucurbitina + dellatorreana)* | 0.29 |
|  |  | Apidae | *Halictus* sp. *(scabiosae + fulvipes)* | 0.01 |
|  |  | Formicidae | *Linepithema humile* | 0.01 |
|  |  | Apidae | *Rhodanthidium septemdentatum* | 0.13 |
|  |  | Syrphidae | *Sphaerophoria* sp. *(scripta + rueppellii)* | 0.05 |
|  |  | Syrphidae | *Syritta pipiens* | 0.04 |
|  |  | Apidae | *Xylocopa violacea* | 0.01 |
| Asteraceae | *Hypochoeris achyrophorus* | Buprestidae | *Anthaxia funerula* | 0.04 |
|  |  | Braconidae | Braconidae sp1 | 0.01 |
|  |  | Bibionidae | *Dilophus antipedalis* | 0.04 |
|  |  | Miridae | *Lepydargyrus ancorifer* | 0.01 |
|  |  | Nitidulidae | *Meligethes* sp. | 0.26 |
|  |  | Mordellidae | *Mordellistena* sp. | 0.04 |
|  |  | Oedemeridae | *Oedemera caudata* | 0.04 |
|  |  | Oedemeridae | *Oedemera flavipes* | 0.03 |
|  |  | Dasytidae | *Psilotrix* sp. *(illustris + cyaneus + aureolus)* | 0.37 |
|  |  | Chrysomelidae | *Spermophagus* sp. | 0.22 |
|  |  | Curculionidae | *Tychius aureolus* | 0.06 |

**Table S2** (*cont*.)

| **PLANT** | | **INSECT POLLINATOR** | | **Interaction frequency** |
| --- | --- | --- | --- | --- |
| **Family** | **Species name** | **Family** | **Species or morphospecies name** |
| Leguminosae | *Lotus corniculatus* | Apidae | *Andrena* sp1 *(ovatula + fulvipes)* | 0.90 |
|  |  | Apidae | *Apis mellifera* | 1.06 |
|  |  | Tachinidae | *Clairvillia pninae* | 0.03 |
|  |  | Apidae | *Colletes abeillei* | 0.34 |
|  |  | Bibionidae | *Dilophus antipedalis* | 0.11 |
|  |  | Apidae | *Hoplitis leucomelans* | 0.20 |
|  |  | Apidae | *Lasioglossum malachurum* | 0.01 |
|  |  | Apidae | *Megachile apicalis* | 0.05 |
|  |  | Apidae | *Megachile pilidens* | 0.10 |
|  |  | Nitidulidae | *Meligethes* sp. | 0.03 |
|  |  | Apidae | *Osmia andrenoides* | 0.05 |
|  |  | Apidae | *Osmia aurulenta* | 0.05 |
|  |  | Apidae | *Osmia caerulescens* | 0.27 |
|  |  | Apidae | *Osmia versicolor* | 0.70 |
|  |  | Lycenidae | *Polyommatus icarus* | 0.34 |
|  |  | Apidae | *Rhodanthidium septemdentatum* | 0.03 |
|  |  | Sarcophagidae | *Sarcophaga villeneuveana* | 0.01 |
|  |  | Chrysomelidae | *Spermophagus* sp. | 0.01 |

**Table S2** (*cont*.)

| **PLANT** | | **INSECT POLLINATOR** | | **Interaction frequency** |
| --- | --- | --- | --- | --- |
| **Family** | **Species name** | **Family** | **Species or morphospecies name** |
| Leguminosae | *Lotus cytisoides* | Apidae | *Andrena* sp1 *(ovatula + fulvipes)* | 0.18 |
|  |  | Apidae | *Apis mellifera* | 1.35 |
|  |  | Apidae | *Bombus terrestris* | 0.01 |
|  |  | Apidae | *Ceratina* sp. *(cucurbitina + dellatorreana)* | 0.06 |
|  |  | Bibionidae | *Dilophus antipedalis* | 0.06 |
|  |  | Formicidae | *Linepithema humile* | 0.01 |
|  |  | Nitidulidae | *Meligethes* sp. | 0.10 |
|  |  | Apidae | *Osmia caerulescens* | 0.02 |
|  |  | Apidae | *Rhodanthidium septemdentatum* | 0.13 |
| Leguminosae | *Medicago littoralis* | Curculionidae | Curculionidae sp1 | 0.01 |
|  |  | Nitidulidae | *Meligethes* sp. | 0.26 |
|  |  | Syrphidae | *Paragus tibialis* | 0.01 |
|  |  | Lycenidae | *Polyommatus icarus* | 0.01 |
|  |  | Tachinidae | *Siphona* sp. | 0.01 |
| Leguminosae | *Melilotus indica* | Apidae | *Andrena* subgen. *Micrandrena* | 0.61 |
|  |  | Apidae | *Andrena* sp1 *(ovatula + fulvipes)* | 1.97 |
|  |  | Apidae | *Andrena* sp4 | 0.45 |
|  |  | Apidae | *Lasioglossum griseolum* | 0.45 |
|  |  | Cantharidae | *Rhagonycha fulva* | 0.45 |
|  |  | Syrphidae | *Sphaerophoria* sp. *(scripta + rueppellii)* | 0.15 |
| Leguminosae | *Melilotus segetalis* | Apidae | *Andrena* sp1 *(ovatula + fulvipes)* | 1.75 |
|  |  | Apidae | *Apis mellifera* | 7.11 |
|  |  | Syrphidae | *Sphaerophoria* sp. *(scripta + rueppellii)* | 0.02 |
| Scrophulariaceae | *Parentucellia viscosa* | Apidae | *Anthophora plumipes* | 0.08 |
|  |  | Apidae | *Lasioglossum griseolum* | 0.03 |

**Table S2** (*cont*.)

| **PLANT** | | **INSECT POLLINATOR** | | **Interaction frequency** |
| --- | --- | --- | --- | --- |
| **Family** | **Species name** | **Family** | **Species or morphospecies name** |
| Rosaceae | *Potentilla reptans* | Apidae | *Andrena* sp1 *(ovatula + fulvipes)* | 0.03 |
|  |  | Apidae | *Apis mellifera* | 0.43 |
|  |  | Blattodea | Blattodea sp. | 0.01 |
|  |  | Chrysomelidae | *Bruchidius* sp1 | 0.01 |
|  |  | Apidae | *Ceratina* sp. *(cucurbitina + dellatorreana)* | 0.01 |
|  |  | Cerambicidae | *Chlorophorus trifasciatus* | 0.02 |
|  |  | Gasteruptiidae | *Gasteruption undulatum* | 0.02 |
|  |  | Apidae | *Hoplitis leucomelans* | 0.07 |
|  |  | Apidae | *Hylaeus pictus* | 0.51 |
|  |  | Apidae | *Lasioglossum griseolum* | 0.03 |
|  |  | Apidae | *Lasioglossum malachurum* | 0.01 |
|  |  | Formicidae | *Linepithema humile* | 0.05 |
|  |  | Nitidulidae | *Meligethes* sp. | 0.01 |
|  |  | Mordellidae | *Mordellistena* sp. | 0.02 |
|  |  | Stratiomyidae | *Nemotelus pantherinus* | 0.03 |
|  |  | Oedemeridae | *Oedemera caudata* | 0.20 |
|  |  | Oedemeridae | *Oedemera flavipes* | 0.05 |
|  |  | Apidae | *Osmia caerulescens* | 0.03 |
|  |  | Apidae | *Osmia versicolor* | 0.17 |
|  |  | Syrphidae | *Paragus tibialis* | 0.05 |
|  |  | Dasytidae | *Psilotrix* sp. *(illustris + cyaneus + aureolus)* | 0.02 |
|  |  | Apidae | *Rhodanthidium septemdentatum* | 0.01 |
|  |  | Chrysomelidae | *Spermophagus* sp. | 0.60 |
|  |  | Syrphidae | *Sphaerophoria* sp. *(scripta+rueppellii)* | 0.65 |
|  |  | Rhinophoridae | *Stevenia deceptoria* | 0.01 |
|  |  | Syrphidae | *Syritta pipiens* | 0.01 |
|  |  | Formicidae | *Tapinoma madeirense* | 0.08 |

**Table S2** (*cont*.)

| **PLANT** | | **INSECT POLLINATOR** | | **Interaction frequency** |
| --- | --- | --- | --- | --- |
| **Family** | **Species name** | **Family** | **Species or morphospecies name** |
| Rosaceae | *Potentilla reptans* | Curculionidae | *Tychius aureolus* | 0.05 |
| Dipsacaceae | *Scabiosa maritima* | - | Acari sp. | 0.17 |
|  |  | Buprestidae | *Acmaoederella discoida* | 0.01 |
|  |  | Apidae | *Apis mellifera* | 5.03 |
|  |  | Crabronidae | *Bembix occulata* | 0.04 |
|  |  | Bombyliidae | *Bombylius posticus* | 0.08 |
|  |  | Apidae | *Bombus terrestris* | 0.02 |
|  |  | Lycaenidae | *Celastrina argiolus* | 0.01 |
|  |  | Apidae | *Ceratina* sp. *(cucurbitina + dellatorreana)* | 0.46 |
|  |  | Pieridae | *Colias croceus* | 0.02 |
|  |  | Apidae | *Halictus* sp. *(scabiosae + fulvipes)* | 2.99 |
|  |  | Syrphidae | *Helophilus trivittatus* | 0.12 |
|  |  | Scoliidae | *Megascolia* sp. | 0.02 |
|  |  | Nitidulidae | *Meligethes* sp. | 0.43 |
|  |  | Mordellidae | *Mordellistena* sp. | 0.03 |
|  |  | Stratiomyidae | *Nemotelus pantherinus* | 0.01 |
|  |  | Oedemeridae | *Oedemera caudata* | 0.04 |
|  |  | Oedemeridae | *Oedemera flavipes* | 0.01 |
|  |  | Oedemeridae | *Oedemera simplex* | 0.01 |
|  |  | Cetoniidae | *Oxythyrea funesta* | 0.01 |
|  |  | Syrphidae | *Paragus tibialis* | 0.01 |
|  |  | Dasytidae | *Psilotrix* sp. *(illustris + cyaneus + aureolus)* | 0.03 |
|  |  | Apidae | *Rhodanthidium septemdentatum* | 0.02 |
|  |  | Syrphidae | *Sphaerophoria* sp. *(scripta + rueppellii)* | 0.02 |
|  |  | Nymphalidae | *Vanessa carduii* | 1.04 |

**Table S2** (*cont*.)

| **PLANT** | | **INSECT POLLINATOR** | | **Interaction frequency** |
| --- | --- | --- | --- | --- |
| **Family** | **Species name** | **Family** | **Species or morphospecies name** |
| Caryophyllaceae | *Silene vulgaris* | Nitidulidae | *Meligethes* sp. | 0.14 |
|  |  | Dasytidae | *Psilotrix* sp. *(illustris + cyaneus + aureolus)* | 0.01 |
|  |  | Nymphalidae | *Vanessa carduii* | 0.03 |
| Labiatae | *Teucrium dunense* | Apidae | *Andrena* sp1 *(ovatula + fulvipes)* | 1.14 |
|  |  | Apidae | *Andrena* sp2 | 0.87 |
|  |  | Apidae | *Andrena* sp3 | 0.54 |
|  |  | Apidae | *Apis mellifera* | 40.82 |
|  |  | Crabronidae | *Bembix occulata* | 3.15 |
|  |  | Apidae | *Ceratina* sp. *(cucurbitina + dellatorreana)* | 0.16 |
|  |  | Cerambicidae | *Chlorophorus trifasciatus* | 0.03 |
|  |  | Apidae | *Colletes dusmeti* | 0.16 |
|  |  | Formicidae | *Crematogaster laestrygon* | 0.01 |
|  |  | Syrphidae | *Eristalinus aeneus* | 0.33 |
|  |  | Syrphidae | *Eristalinus megacephalus* | 0.03 |
|  |  | Apidae | *Halictus* sp. *(scabiosae + fulvipes)* | 0.57 |
|  |  | Apidae | *Heriades rubicolus* | 0.09 |
|  |  | Apidae | *Lasioglossum gemmeus* | 0.55 |
|  |  | Apidae | *Lasioglossum malachurum* | 0.16 |
|  |  | Formicidae | *Linepithema humile* | 0.71 |
|  |  | Scoliidae | *Megascolia hortorum* | 0.58 |
|  |  | Scoliidae | *Megascolia* sp. | 3.21 |
|  |  | Nitidulidae | *Meligethes* sp. | 0.10 |
|  |  | Crabronidae | *Philanthus triangulum* | 0.16 |
|  |  | Lycenidae | *Polyommatus icarus* | 0.20 |
|  |  | Vespidae | *Polistes* sp. *(dominulus + gallicus)* | 0.30 |
|  |  | Sphecidae | *Prionyx kirbii* | 1.25 |

**Table S2** (*cont*.)

| **PLANT** | | **INSECT POLLINATOR** | | **Interaction frequency** |
| --- | --- | --- | --- | --- |
| **Family** | **Species name** | **Family** | **Species or morphospecies name** |
| Labiatae | *Teucrium dunense* | Apidae | *Rhodanthidium septemdentatum* | 0.18 |
|  |  | Syrphidae | *Sphaerophoria* sp. *(scripta + rueppellii)* | 0.17 |
|  |  | Calliphoridae | *Stomorhina lunata* | 0.11 |
|  |  | Syrphidae | *Syritta pipiens* | 0.16 |
|  |  | Nymphalidae | *Vanessa carduii* | 1.25 |
| Scrophulariaceae | *Verbascum sinuatum* | Apidae | *Ceratina* sp. *(cucurbitina + dellatorreana)* | 0.03 |
|  |  | Apidae | *Ceylalictus variegatus* | 0.05 |
|  |  | Syrphidae | *Eristalinus aeneus* | 0.02 |
|  |  | Apidae | *Hylaeus pictus* | 0.02 |
|  |  | Formicidae | *Linepithema humile* | 0.50 |
|  |  | Curculionidae | *Mogulones* sp. | 0.01 |
|  |  | Cetoniidae | *Oxythyrea funesta* | 0.01 |
|  |  | Syrphidae | *Paragus tibialis* | 0.01 |
|  |  | Syrphidae | *Sphaerophoria* sp. *(scripta + rueppellii)* | 0.05 |
|  |  | Calliphoridae | *Stomorhina lunata* | 0.03 |
|  |  | Syrphidae | *Syritta pipiens* | 0.01 |
