# Supplementary material for: Linking Plant Specialization to Dependence in Interactions for Seed Set in Pollination Networks
Source: PLoS One. 2013 Oct 30;8(10):e78294. doi: 10.1371/journal.pone.0078294 (PMC3813576; doi:10.1371/journal.pone.0078294)
Supplement: Table S3 — List of plant-pollinator interactions observed in PM site for the 11 selected species. Interaction frequency is the number of visits per flower per unit time made by each insect pollinator species. (DOC) [file pone.0078294.s004.doc]

**Table S3.**

| **PLANT** | | **INSECT POLLINATOR** | | **Interaction frequency** |
| --- | --- | --- | --- | --- |
| **Family** | **Species name** | **Family** | **Species or morphospecies name** |
| Caryophyllaceae | *Arenaria grandiflora* | Anthomyiidae | *Adia cinerella* | 0.01 |
|  |  | Bruchidae | *Bruchidius* sp. | 0.03 |
|  |  | Syrphidae | *Eristalis tenax* | 0.05 |
|  |  | Chrysididae | *Holopyga fervida* | 0.03 |
|  |  | Oedemeridae | *Oedemera flavipes* | 0.01 |
|  |  | Apidae | *Osmia latreillei* | 0.04 |
|  |  | Syrphidae | *Paragus pecciolii* | 0.04 |
|  |  | Sarcophagidae | *Sarcophaga nigriventis* | 0.03 |
| Asteraceae | *Bellium bellidioides* | Anthomyiidae | *Anthomyia pluvialis* | 0.01 |
|  |  | Malachiidae | *Attalus* sp. | 0.01 |
|  |  | Braconidae | *Chelonus* sp. | 0.07 |
|  |  | Tachinidae | *Cylindromyia brassicaria* | 0.04 |
|  |  | Tachinidae | *Gymnosoma* sp. | 0.01 |
|  |  | Apidae | *Lasioglossum transitorium planulum* | 0.01 |
|  |  | Sphingidae | *Macroglossum stellatarum* | 0.01 |
|  |  | Oedemeridae | *Oedemera flavipes* | 0.05 |
|  |  | Syrphidae | *Paragus pecciolii* | 0.02 |
|  |  | Syrphidae | *Paragus tibialis* | 0.07 |
|  |  | Lycaenidae | *Polyommatus icarus* | 0.01 |
|  |  | Chloropidae | *Polyodaspis sulcicollis* | 0.01 |
|  |  | Syrphidae | *Sphaerophoria* sp. | 0.04 |

**Table S3** (*cont*.)

| **PLANT** | | **INSECT POLLINATOR** | | **Interaction frequency** |
| --- | --- | --- | --- | --- |
| **Family** | **Species name** | **Family** | **Species or morphospecies name** |
| Asteraceae | *Carlina corymbosa* | Apidae | *Andrena* sp. | 0.04 |
|  |  | Apidae | *Apis mellifera* | 1.25 |
|  |  | Malachiidae | *Attalus* sp. | 0.01 |
|  |  | Curculionidae | *Baris* sp. | 0.01 |
|  |  | Syrphidae | *Chrysotoxum intermedium* | 0.03 |
|  |  | Syrphidae | *Eristalinus taeniops* | 0.03 |
|  |  | Syrphidae | *Eristalis tenax* | 0.01 |
|  |  | Syrphidae | *Eupeodes corollae* | 0.05 |
|  |  | Apidae | *Halictus scabiosae* | 0.26 |
|  |  | Apidae | *Lasioglossum nitidulum hammi* | 0.01 |
|  |  | Apidae | *Megachile pilidens* | 0.01 |
|  |  | Mordellidae | *Mordellistena* sp. | 0.03 |
|  |  | Lycaenidae | *Polyommatus icarus* | 0.03 |
|  |  | Apidae | *Rhodanthidium septemdentatum* | 0.03 |
|  |  | Calliphoridae | *Stomorhina lunata* | 0.20 |
|  |  | Bombyliidae | *Villa hottentotta* | 0.03 |
|  |  | Bombyliidae | *Villa* sp. | 0.01 |
|  |  | Apidae | *Xylocopa violacea* | 0.01 |

**Table S3** (*cont.*)

| **PLANT** | | **INSECT POLLINATOR** | | **Interaction frequency** |
| --- | --- | --- | --- | --- |
| **Family** | **Species name** | **Family** | **Species or morphospecies name** |
| Asteraceae | *Crepis triasii* | Malachiidae | *Attalus* sp. | 0.04 |
|  |  | Apidae | *Ceratina cucurbitina* | 0.01 |
|  |  | Braconidae | *Chelonus* sp. | 0.01 |
|  |  | Syrphidae | *Eristalis tenax* | 0.07 |
|  |  | Apidae | *Halictus fulvipes* | 0.01 |
|  |  | Apidae | *Halictus vestitus* | 0.07 |
|  |  | Tenebrionidae | *Isomira* sp. | 0.01 |
|  |  | Apidae | *Lasioglossum nitidulum hammi* | 0.01 |
|  |  | Oedemeridae | *Oedemera flavipes* | 0.14 |
|  |  | Apidae | *Osmia latreillei* | 0.01 |
|  |  | Bombyliidae | *Phthiria pulicaria* | 0.01 |
|  |  | Bombyliidae | *Phthiria* sp. | 0.02 |
|  |  | Formicidae | *Plagiolepis pygmaea* | 0.04 |
|  |  | Apidae | *Rhodanthidium septemdentatum* | 0.04 |
| Rubiaceae | *Galium balearicum* | Braconidae | *Chelonus* sp. | 0.09 |
| Rubiaceae | *Galium cinereum* | Mordellidae | *Mordellistena* sp. | 0.02 |
|  |  | Oedemeridae | *Oedemera flavipes* | 0.14 |
| Cistaceae | *Helianthemum apenninum* | Pieridae | *Colias croceus* | 0.01 |
|  |  | Oedemeridae | *Oedemera flavipes* | 0.09 |
|  |  | Syrphidae | *Sphaerophoria* sp. | 0.02 |
|  |  | Formicidae | *Temnothorax specularis* | 0.02 |

**Table S3** (*cont.*)

| **PLANT** | | **INSECT POLLINATOR** | | **Interaction frequency** |
| --- | --- | --- | --- | --- |
| **Family** | **Species name** | **Family** | **Species or morphospecies name** |
| Labiatae | *Rosmarinus officinalis* | Apidae | *Apis mellifera* | 1.60 |
|  |  | Apidae | *Ceratina cucurbitina* | 0.11 |
|  |  | Syrphidae | *Eristalis tenax* | 0.02 |
|  |  | Apidae | *Eucera oraniensis* | 0.02 |
|  |  | Syrphidae | *Helophilus trivittatus* | 0.02 |
|  |  | Sphingidae | *Macroglossum stellatarum* | 0.13 |
|  |  | Formicidae | *Plagiolepis pygmaea* | 0.02 |
|  |  | Melyridae | *Psilotrix illustris* | 0.02 |
|  |  | Nymphalidae | *Vanessa cardui* | 0.07 |
| Asteraceae | *Santolina chamaecyparissus* | Dermestidae | *Anthrenus pimpinellae* | 0.01 |
|  |  | Malachiidae | *Attalus* sp. | 0.01 |
|  |  | Syrphidae | *Chrysotoxum intermedium* | 0.02 |
|  |  | Tachinidae | *Cylindromyia brassicaria* | 0.15 |
|  |  | Tachinidae | *Dionaea aurifrons* | 0.01 |
|  |  | Syrphidae | *Eristalis tenax* | 0.06 |
|  |  | Apidae | *Lasioglossum transitorium planulum* | 0.07 |
|  |  | Apidae | *Megachile pilidens* | 0.01 |
|  |  | Mordellidae | *Mordellistena* sp. | 0.19 |
|  |  | Oedemeridae | *Oedemera flavipes* | 0.52 |
|  |  | Syrphidae | *Paragus tibialis* | 0.12 |
|  |  | Bombyliidae | *Phthiria pulicaria* | 0.09 |
|  |  | Bombyliidae | *Phthiria* sp. | 0.02 |
|  |  | Chloropidae | *Polyodaspis sulcicollis* | 0.08 |
|  |  | Calliphoridae | *Stomorhina lunata* | 0.09 |

**Table S3** (*cont.*)

| **PLANT** | | **INSECT POLLINATOR** | | **Interaction frequency** |
| --- | --- | --- | --- | --- |
| **Family** | **Species name** | **Family** | **Species or morphospecies name** |
| Crassulaceae | *Sedum dasyphyllum* | Apidae | *Halictus vestitus* | 0.01 |
|  |  | Apidae | *Hoplitis leucomelans* | 0.03 |
|  |  | Apidae | *Lasioglossum nitidulum hammi* | 0.07 |
|  |  | Apidae | *Lasioglossum transitorium planulum* | 0.21 |
|  |  | Oedemeridae | *Oedemera flavipes* | 0.03 |
|  |  | Chloropidae | *Oscinella frit* | 0.03 |
|  |  | Bombyliidae | *Phthiria pulicaria* | 0.03 |
|  |  | Formicidae | *Plagiolepis pygmaea* | 0.13 |
| Labiatae | *Teucrium asiaticum* | Apidae | *Amegilla quadrifasciata* | 0.40 |
|  |  | Apidae | *Anthidium cingulatum* | 0.01 |
|  |  | Apidae | *Anthidium manicatum* | 0.09 |
|  |  | Apidae | *Apis mellifera* | 0.41 |
|  |  | Syrphidae | *Chrysotoxum intermedium* | 0.01 |
|  |  | Apidae | *Halictus vestitus* | 0.02 |
|  |  | Apidae | *Lasioglossum nitidulum hammi* | 0.09 |
|  |  | Sphingidae | *Macroglossum stellatarum* | 0.39 |
|  |  | Apidae | *Megachile pilidens* | 0.52 |
|  |  | Apidae | *Protosmia minutula* | 0.01 |
|  |  | Apidae | *Rhodanthidium septemdentatum* | 0.04 |
|  |  | Bombyliidae | *Villa* sp. | 0.01 |
|  |  | Apidae | *Xylocopa violacea* | 0.01 |
